# *Lymantria dispar* (L.) (Lepidoptera: Erebidae): Current Status of Biology, Ecology, and Management in Europe with Notes from North America

**DOI:** 10.3390/insects13090854

**Published:** 2022-09-19

**Authors:** Maria C. Boukouvala, Nickolas G. Kavallieratos, Anna Skourti, Xavier Pons, Carmen López Alonso, Matilde Eizaguirre, Enrique Benavent Fernandez, Elena Domínguez Solera, Sergio Fita, Tanja Bohinc, Stanislav Trdan, Paraskevi Agrafioti, Christos G. Athanassiou

**Affiliations:** 1 Laboratory of Agricultural Zoology and Entomology, Department of Crop Science, Agricultural University of Athens, 75 Iera Odos Str., 11855 Athens, Greece; 2 Department of Crop and Forest Sciences, Agrotecnio Centre, Universitat de Lleida, Av Rovira Roure 191, 25198 Lleida, Spain; 3 AIMPLAS, Plastics Technology Centre, València Parc Tecnològic, Gustave Eiffel 4, 46980 Paterna, Spain; 4 Department of Agronomy, Biotechnical Faculty, University of Ljubljana, Jamnikarjeva 101, 1000 Ljubljana, Slovenia; 5 Laboratory of Entomology and Agricultural Zoology, Department of Agriculture, Crop Production and Rural Environment, University of Thessaly, Phytokou Str., 38446 Nea Ionia, Greece

**Keywords:** European spongy moth, spread, losses, outbreaks, health issues, control

## Abstract

**Simple Summary:**

In the current review, we gathered and summarized the up-to-date information on the life cycle, distribution, outbreaks, control, and health issues to humans and animals of the European Spongy moth. Overall, this noxious species is easily expanded to new areas, causing serious large-scale damage rapidly. The management of this insect is difficult since the chemicals are harmful to human health and the environment, and natural enemies are not able to cause sufficient reduction of the populations of *L. dispar*. Finally, the potential use of biotechnological and physical methods against *L. dispar* is discussed.

**Abstract:**

The European Spongy moth, *Lymantria dispar* (L.) (Lepidoptera: Erebidae), is an abundant species found in oak woods in Central and Southern Europe, the Near East, and North Africa and is an important economic pest. It is a voracious eater and can completely defoliate entire trees; repeated severe defoliation can add to other stresses, such as weather extremes or human activities. *Lymantria dispar* is most destructive in its larval stage (caterpillars), stripping away foliage from a broad variety of trees (>500 species). Caterpillar infestation is an underestimated problem; medical literature reports that established populations of caterpillars may cause health problems to people and animals. Inflammatory reactions may occur in most individuals after exposure to setae, independent of previous exposure. Currently, chemical and mechanical methods, natural predators, and silvicultural practices are included for the control of this species. Various insecticides have been used for its control, often through aerial sprayings, which negatively affect biodiversity, frequently fail, and are inappropriate for urban/recreational areas. However, bioinsecticides based on various microorganisms (e.g., entomopathogenic viruses, bacteria, and fungi) as well as technologies such as mating disruption using sex pheromone traps have replaced insecticides for the management of *L. dispar*.

## 1. Introduction

**Figure 1:**
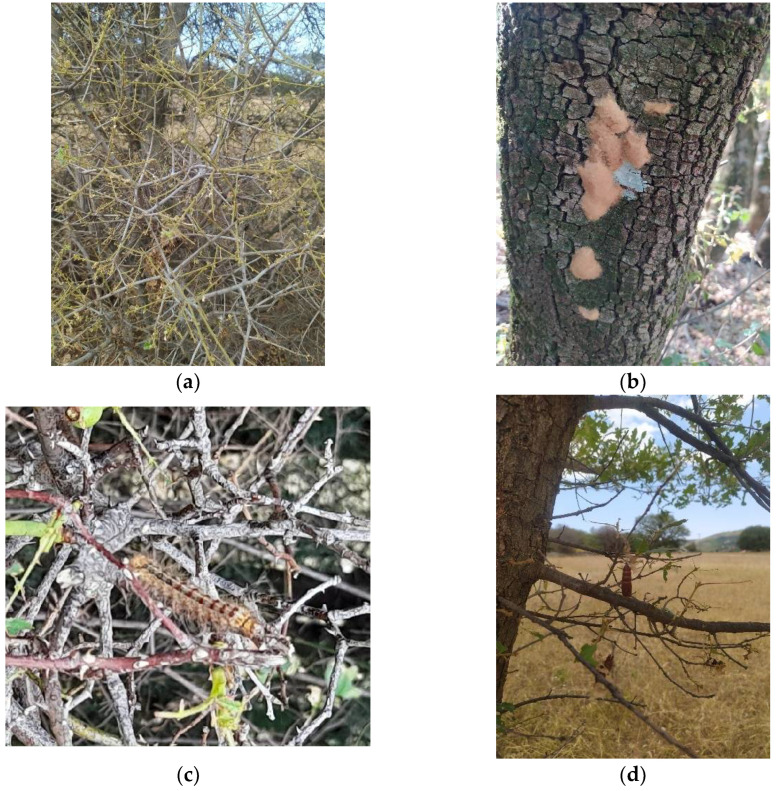

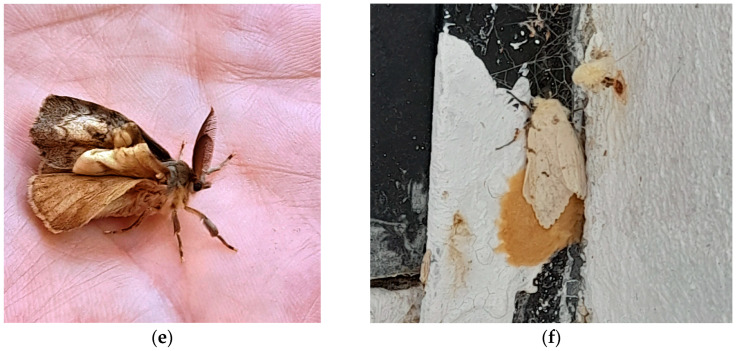
(**a**) Severe defoliation of oak by *Lymantria dispar*. (**b**) Egg clusters of *L. dispar*. (**c**) Late instar larva of *L. dispar*. (**d**) Pupa of *L. dispar*. (**e**) Newly emerged adult male of *L. dispar*. (**f**) Female of *L. dispar* laying eggs on a wall.

Sporadic outbreaks of insect pests are a major challenge to forest health in many countries, leading to serious environmental resource losses and degradations. One of these pests is the spongy moth (formerly known as gypsy moth) *Lymantria dispar* (L.) (Lepidoptera: Erebidae), a notorious invasive polyphagous pest, causing widespread loss of leaves in forests in Europe, Asia, North America, and parts of Africa (Figure 1a) [1]. This species recently underwent a change to the common name since the word “gypsy” was considered to be an ethnic slur and now refers to the spongy egg masses [2]. It is also considered very harmful to orchards and urban environments [3,4,5]. In the species name of the *L. dispar*, “dispar” is derived from the Latin word that means “to separate”, depicting the conspicuous sexual dimorphism of this species [6]. Females are larger and more brightly coloured than males and do not fly even though they are winged [7]. The International Union for Conservation of Nature (IUCN) has categorized *L. dispar* as one of the 100 worst invaders globally [8]. According to the European and Mediterranean Plant Protection Organization [9], in the United States of America, *L. dispar* has been a quarantine pest since 1989, while in Europe it has been classified in the A1 list in Azerbaijan and Georgia since 2007 and 2018, respectively, and in the A2 list in Russia since 2014.

*Lymantria dispar* is a univoltine species with four distinct life stages: egg, larva (caterpillar), pupa, and adult (Figure 1b–f) [10,11]. More specifically, the front and vertex of the male’s head is pale gray to light brown; the antennae are bipectinates; and the shaft is completely scaled, with a light brown color and some fuscous scales apically. The forewing is 14.5–22 mm long [12] and is color brown. The hindwings are reddish-brown with a dark brown marginal band and light brown fringe [12]. The front and vertex of the female’s head is white, the scape is speckled white and brown, the antenna has short pectinations, the shaft and pectinations are fuscous, and the apex is white. The length of forewings in females is 20–30 mm [12], being the color white. The hindwing is white, has a discal spot faint, and has a V-shaped at end of the discal cell [12]. Egg clusters are teardrop shaped, their color is yellowish to brownish, are felt-like in appearance, and turn lighter in color with age. Normally, all individual eggs are hidden by the hairs of the female abdomen [12]. First instar larvae are about 3 mm long [6], are very light in weight, and their color is dark brown to black, while the later instar larvae are more colorful [13,14]. The final larval instar is sometimes up to 90 mm long [6]. Full-grown larvae are hairy and have on the dorsum two rows of blue tubercles on the first five segments and two rows of red tubercles on the following six segments [14]. Pupae are dark brown and teardrop shaped, without a silky cocoon. Female pupae are 15–35 mm long, while males are usually smaller than females with a length of about 15–20 mm [13].

The classification of *L. dispar* has been controversial in the past, but it is now generally accepted that the European spongy moth, *Lymantria dispar dispar* L., differs from the Asian spongy moth [15]. Indeed, apart from the European spongy moth, which is spread throughout Europe and can be found in all environmental zones, with the exception of the Alpine North and part of the Boreal zone [16], two other subspecies have been recognized: (i) *L. dispar asiatica* Vnukovskij, which is found mostly in China, in Korea, and in the east Ural Mountains, and (ii) *L. dispar japonica* Motschulsky, which occurs on the main islands of Japan [12]. However, all three subspecies are polyphagous [17,18], with *L. dispar asiatica* having a wider range of host plants than *L. dispar dispar* [15,19,20,21,22,23,24,25,26]. In addition, females of both Asian subspecies are capable of flight, in contrast to females of the European subspecies that are flightless [10,12,23,27,28], a fact that may impede the spread of the latter subspecies. However, females of the European subspecies have little to no selectivity during oviposition and they can deposit egg masses on available substrates than can be used as vehicles for the spread of this subspecies [29]. Similarly, females of *L. dispar asiatica* that are able to fly are more attracted to lights, especially at well-lit ports, where they lay eggs on ships, which greatly facilitates their dispersal [30,31]. Recently, several molecular tools based on the mitochondrial barcode region have been used for the identification of the three subspecies. It has been found that nucleotide substitutions within regions of mitogenomes of *L. dispar* may be a useful tool to distinguish among the subspecies and/or detect geographical origins [32].

Populations of *L. dispar* usually remain at low densities. However, under some circumstances that are not well understood, densities can increase rapidly and after few generations achieve pest status. It has been reported that the length of the non-outbreak periods may be linked to climate, being shorter in dry forests and longer in wet ones [33]. The projected increase in temperature will strongly affect the ecology and distribution of exothermic organisms and is likely to trigger outbreaks of certain insect pests [34]. Indeed, the increase in mean temperature is expected to have a positive effect on *L. dispar* dispersal in the more northern ecosystems. Moreover, apart from climate change, inadvertent transportation will help this species to colonize new northern European habitats, as has happened in North America [35].

Currently, management of *L. dispar* is mainly based on biopesticides [36,37]. Moreover, several biocontrol agents have been used for the control of *L. dispar* such as entomopathogenic microorganisms alone or in combinations [36,38,39,40,41,42,43,44,45,46,47,48]. Although parasitoids represent appropriate biological control agents [49], they have not been used very often in IPM programs for controlling *L. dispar* in Europe.

Due to a lack of review studies concerning *L. dispar* in Europe, we aimed to gather and present in detail the up-to-date information from the international bibliography, scientific databases, and journals on the following aspects: description of each life stage, lifespan, preferred plant species, distribution, damages and outbreaks, and various control and monitoring methods (i.e., traps, mating disruption, natural enemies, biological and chemical control). In addition to the considerable damage to forests, *L. dispar* larvae are an underestimated problem; established populations of larvae may cause health problems to people and animals. Thus, special emphasis will be given to the health problems and risks arising from the presence of *L. dispar* larvae in forests and urban/recreational areas for humans and the environment.

## 2. Biology

The lifespan of *L. dispar* adults is about a week, and male emergence usually takes place 1 or 2 days before the females [6]. Specifically, females die about one day after oviposition, whereas males survive for about a week, after mating with different females [11]. *Lymantria dispar* can mate and oviposit soon after adults emerge [10,12,50]. Moreover, females can attract males and mate within two hours of pupal eclosion [51,52]. The courtship behavior is very simple, a male approaches a female, and mating occurs only when the female lifts her wings to allow mating. Copulation lasts for up to an hour, but usually the passage of the spermatophore or sperm packet is accomplished in the first 10 min. After the termination of the sexual interaction, females begin to lay eggs [6,53,54]. Females mate once while males usually mate a few times [55]. Thus, since males are polygamous, almost all females are fertilized, even if males comprise only 17% of the total population [56]. The reproductiive timespan of females is shorter than a week. By the third day after the female’s emergence, their attractiveness decreases significantly, perhaps due to a depletion of the pheromone supply. Thus, there is little likelihood a female will mate if a mate has not been selected by then [6].

Mated females lay eggs on the tree trunks usually at less than 1 m from their pupation site because they are functionally flightless [12,23,27,28,57,58,59]. However, females that have not mated lay eggs that do not hatch and are usually scattered singly or in small, disorganized clusters [39]. Eggs are deposited in clutches directly on the bark or branches of host trees in late summer [12,60,61,62,63,64]. If a female is interrupted during the oviposition, she is able to initiate a new cluster [6]. In addition to host plants, eggs can be found on rocks or other immovable objects (e.g., firewood, recreational vehicles, Christmas trees, boat or cargo containers) and can overwinter there for eight to nine months [10,65]. Generally, females oviposit in the cooler or shady parts of the tree [12]. A female can lay from less than 100 to over 1000 eggs. Under optimal conditions, the mean number of eggs in a cluster is about 750, while at the end of an outbreak when the population begins to decline, it is about 300 [6]. For instance, in Spain, the mean number of eggs per cluster over the entire generation is 250–500 [57,58,59,66,67]. In addition, the egg clusters are covered with hair-like setae from the female’s abdomen to protect them for overwintering [44]. The hairs are long, straight, and generally conical at both ends [68]. Eggs can withstand temperatures below −30 °C (down to −31.7 °C), provided they are deposited in a protected area or insulated with snow [10]. The egg clusters are approximately 3.8 cm long and 1.9 cm wide, whereas the presence of smaller egg clusters indicates that the population of *L. dispar* is declining [69].

The first three weeks of the egg stage consist of embryonation followed by diapause, which is affected by cold and warm conditions [70]. A significant number of studies have been conducted on climatic factors that affect the three stages of diapause (i.e., pre-diapause, diapause, and post-diapause), revealing a complex, non-linear relationship between temperature and progress through the completion of diapause [6,71,72]. The development of the embryo is favored by higher temperatures, completing the prediapause phase in approximately 16 days at 25 °C, but at a lower temperature, this phase takes longer to complete (e.g., 48 d at 15 °C) [73]. This phase is characterized by high respiration rates and abundant morphological development of the embryo [60,73]. The well-developed embryo then enters the next phase, the diapause, as a fully differentiated pharate larva, which lasts several months [60,74], but the role of the temperature is crucial, since at low temperatures, diapause is terminated quickly [60,72,75,76,77]. This phase is characterized by low respiration and developmental rates favored by low temperatures [72]. After exposure to low temperatures to terminate the diapause, elevated temperatures affect the hatching, where at 25 °C, hatching is completed in 11–18 days, while at 15 °C, more time is needed (i.e., 14–25 days), and the respiration rates are again high during the postdiapause phase [78]. There is no evidence that the photoperiod can control diapause, but 60–150 days of low temperature exposure is required [77].

Egg hatching takes place in early spring to mid-May [10]. However, the temperature strongly affects the hatching and activity of new larvae. Most of them hatch within a week, while hatching of egg clusters deposited in cooler areas or at higher altitudes can extend up to a month [6]. Larvae overwinter as embryos protected in eggshells and emerge in spring [12]. Male larvae usually go through five instars, while females go through six instars [63]. However, it has been reported that larvae may go through as many as 11 instars before pupation [70]. If the newly hatched larvae emerge at temperatures lower than 7 °C, they remain on or near the egg cluster [6]. Heavy rainfall during egg hatch may result in drowning of larvae. Moreover, during rainy weather, the first-instar larvae may delay their migration and accumulate on the underside of leaves [79].

After hatching, the larvae feed on the foliage of the host plants for a period of six to eight weeks [10]. First instar larvae begin feeding by cutting small holes in the surface of leaves, whereas later instars feed on the edge of leaves [6]. A distinct change in the feeding rhythm of *L. dispar* occurs as the larvae develop and mature [80]. The larvae are positively phototropic and negatively geotropic when they leave the egg and spin a silk thread as they move [6]. In addition, phototropic changes shift their behavior, with early instar larvae strongly attracted to light and late-instar larvae indifferent or repelled by light [81]. Early instar larvae (typically first- to third-instar) feed on host leaves during the day and stay on the underside of foliage during the evening [61,82]. Late-instar larvae (fourth- to sixth-instar) feed on the canopy overnight, whereas at daybreak, they move downward in search of cryptic resting places (e.g., bark flaps or crevices, litter under the host tree) [52] as these sites provide protection from predators [83,84]. During the diurnal period of larval movement, it is very common for the late instar larvae to abandon the tree they were feeding on and move to a new one [85]. This is a mechanism that allows late instar larvae to use a wider range of plants than early instar larvae [86]. However, in elevated populations, such as populations of more than 1250 egg masses/ha, all larvae feed in the day and night and do not need to hide in a shelter [87]. Approximately 80% of defoliation is conducted by the fifth and sixth instar larvae [88]. In Central Europe, the larvae begin to feed in late April and continue for up to 10 weeks, until they pupate in late June–early July [89].

The larvae stop feeding just before the prepupal stage, which lasts only for about 2 days, where the larvae void the gut, surround themselves in a sparse silken net, and begin to contract in length. The prepupae remain relatively quiescent inside the silken net [6]. Pupation occurs in hidden places such as the underside of branches, cracks in bark, trunk crevices, and under stones or trunks on the soil [57,58,59]. The pupal stage lasts 7–14 days depending on climate and sex. Usually, males emerge a few days earlier than females [50,85].

*Lymantria dispar* adults do not feed, and although moisture is imbibed, their digestive system is not functional [6]. Τhe larvae are the most destructive life stage, as they can feed on a wide range of hosts [17,18,50,90,91,92,93]. In addition, voracious larvae can feed on host trees for up to 10 weeks [94]. The consumption of oak foliage by larvae is about 10 mg for each mg gain in larval biomass [95]. Each larva in a dense population is able to consume about 1270 mg of foliage (dry weight) and reach a weight of 114 mg. This is about 170 cm^2^ foliage corresponding to three red oak leaves. However, a large (750 mg) female larva typical of a sparse population could consume a total of 1000 cm^2^ of foliage [50]. Thus, the sixth instar female larvae are considered the most ravenous feeders and have often twice the size of full-grown male larvae [11].

Forest tree species can be categorized as “susceptible”, “resistant”, or “immune” to defoliation by *L. dispar* larvae [17,96]. “Susceptible” tree species are those that are consumed by all larval instars; “resistant” are the species that only some larval instars consume, or when susceptible species are not available (Table 1); and “immune” species are rarely, if ever, consumed by any larval instar. Some tree species are considered resistant to *L. dispar* larvae unless in close proximity to susceptible species. In the case that a preferred tree species is not available, the larvae may alternatively feed on the red and sugar maple, *A. rubrum* L. and *A. saccharum* Marshall (Sapindales: Sapindaceae); the American beech, *Fagus grandifolia* Ehrh. (Fagales: Fagaceae); and the American elm, *Ulmus americana* L. (Rosales: Ulmaceae) [10,17].

In Europe, the preferred hosts vary by region, but include species of *Quercus*, *Carpinus*, *Alnus*, *Prunus*, *Populus*, *Gleditsia*, *Tilia*, *Corylus*, and *Robinia* [63,97,98]. For instance, in Lithuania, which is at the northern limits of its range, species of birch (*Betula*) and alder (*Alnus*) are the primary hosts, while in Spain, Portugal, and Sardinia, the cork oak, *Quercus suber* L. (Fagales: Fagaceae), is the dominant host, and stands of this species have been frequently defoliated. The distribution of *L. dispar* in the rest of Europe is associated with the presence of up to seven species of *Quercus*, especially the Austrian oak, *Q. cerris* L.; the pedunculate oak, *Q. robur* L.; and the sessile oak, *Q. petraea* (Matt.) Liebl. (Fagales: Fagaceae), but this latter species is less preferred among the Central European oaks [98]. The hornbeam, *Carpinus betulus* L. (Fagales: Betulaceae), is usually mixed in stands of oaks in Central Europe and is considered an equally preferred host along with species of *Populus*, *Alnus*, and *Salix* [98,99]. In southern France and the Balkans, *Q. suber*, *Q. pubescens*, and *Q. ilex* serve as the main hosts. However, there is an exception to the close relationship between oak species and *L. dispar* populations in the Danube delta of Romania, where 27,000 ha of *Populus* and *Salix* stands serve as preferred hosts for *L. dispar* larvae [98].

In North America, *L. dispar* feed on >300 species of woody plants but prefer the species *Quercus*, *Salix*, *Populus*, and *Betula* [17]. More specifically, 148 host trees have been identified as highly susceptible hosts out of a total of 449 tree species that larvae can feed on [17]. *Quercus* and *Salix* are the most susceptible genera, with the remaining highly susceptible species coming from 28 other tree genera including *Larix*, a deciduous conifer [100]. In both Europe and North America, the variety of hosts plants the larvae utilize expands when they reach the fourth instar, including several conifers, such as some species of pine, spruce, and hemlock [101].

It has been reported that early instar larvae are not able to complete their development on non-deciduous conifers [18,102,103]. However, in outbreak mode, larvae will consume coniferous foliage, and these trees tend to be much less resistant to defoliation and may die after a single attack [104]. On the other hand, hardwood trees that are initially in good condition can produce new leaves after a *L. dispar* attack (which generally takes place in June and early July) and can often withstand several years of defoliation without dying [104]. Consequently, in the case of mixed pine–hardwood stands such as those of eastern North America, defoliation by larvae is largely limited to hardwood hosts, and outbreaks generally do not occur in stands in which the proportion of oaks or other susceptible host plant species is less than 20% of host basal area [105,106].

In Spain, *L. dispar* has been reported on *Quercus* sp., *Castanea* sp., *Corylus* sp., and *Fagus* sp. but also on *Populus* sp., *Ulmus* sp., *Arbutus* sp., *Prunus* sp., *Acer* sp., *Salix* sp., *Betula* sp., *Alnus* sp., or *Pinus* sp. It has been reported to damage the strawberry tree, *Arbutus unedo* L. 1753 (Ericales: Ericaceae), and coniferous species, such as the Aleppo pine; *Pinus halepensis* Mill., in the Balearic Islands and Catalonia; the maritime pine, *Pinus pinaster* Aiton, in central Spain and the Balearic Islands; and the radiata pine, *Pinus radiata* D.Don (Pinales: Pinaceae), in northern Spain [58,66]. This forms an important threat to public health as pine trees constitute a major species in urban/suburban environments and cover large forest areas in Southern Europe, while their presence is common in schools and other public areas, potentially affecting susceptible individuals, such as children.

It has been found in the provinces of Pontevedra (north-western Spain) and Asturias (northern Spain) in the outbreaks during 1952–1953 [107,108]. In northwestern Portugal, 290 ha of a 15-year-old plantation of *P. radiata* were severely defoliated in 1991 [109]. Moreover, Miller and Hanson [110], conducting laboratory tests, found that the first instar larvae originated from egg clusters collected in Oregon, USA, completed their development on different coniferous tree species including the white fir, *Abies concolor* (Gordon) Lindley ex Hildebrand; the blue spruce, *Picea pungens* Engelm.; the ponderosa pine, *Pinus ponderosa* Douglas ex C.Lawson; and the Douglas fir, *Pseudotsuga menziesii* (Mirbel) Franco (Pinales: Pinaceae). More recently, Castedo-Dorado et al. [111] reported that the *L. dispar* was able to complete development in the field and severely defoliated trees of *P. radiata* in Spain. Radiata pine is native to California, but has spread widely elsewhere, especially in the southern hemisphere, due to its planting for commercial forestry. In 1950, extensive planting of this species began in the Northern Iberian Peninsula [112]. Consequently, the presense of *P. radiata* in this region, along with other southern European countries, are the only areas where both radiata pine and *L. dispar* overlap, since radiata pine is absent from *L. dispar*’s invasion area in North America and the insect pest is absent in the southern hemisphere [111].

## 3. Ecology of *L. dispar*

### 3.1. Distribution

*Lymantria dispar* is a serious insect pest in southern and central Europe [34]. It was intentionally introduced to the east coast of North America from Western Europe in 1869 for silk production [12,50,52,113,114,115,116]. Professor L. Trouvelot maintained colonies of this species, attempting to reproduce better silkworm larvae by crossing them with the native silkworm, *Antheraea polyphemus* (Cramer) (Lepidoptera: Saturniidae), but a small number of *L. dispar* larvae escaped from his laboratory [98,115,117]. This species has been established there, reaching nearly the middle of the continent [118]; it has spread throughout New England and the adjoining provinces of Canada. The peak of the infestation has reached Maine, Wisconsin, Illinois, Indiana, Ohio, West Virginia, Virginia, North Carolina, Ontario, Quebec, New Brunswick, and Nova Scotia [27,119]. *Lymantria dispar* has become a much more serious pest in the United States than in its native regions [6,113,115,120,121,122]. *Lymantria dispar* has recently expanded to several ecosystems globally, where it was previously nonnative, due to high vehicle traffic, international shipping, and global warming, which favors its winter survival and enhances its feeding activity in northern regions, as shown both in research simulations and empirically [35].

*Lymantria dispar* is widely distributed in Europe, occurring in many environments of this continent, with the exception of Alpine North and partially the Boreal zone [9,16]. It is also found from latitude 60° N in mid-Scandinavia to 35° N on the Mediterranean coast and occurs from the Mediterranean scrub land to the temperate deciduous forests [118]. The northern limit proceeds through southern Sweden and Finland and descends from about 60° to 50° lines of latitude through Europe and Russia. The southern limit begins in the west in northern Morocco, Algeria, and Tunisia and proceeds east to include all of the Mediterranean islands, on a line through Israel into Asia [98]. The latitudinal distribution of *L. dispar* in Canada and the United States lies between 35° N and 48° N [91]. Generally, damage caused by *L. dispar* in Europe increases from west to east and from north to south [123]. Furthermore, due to the projected climate change, *L. dispar* may be a threat to more northerly ecosystems [35]. 

The spread of *L. dispar* in Europe has been recorded in the database of the European and Mediterranean Plant Protection Organization [9]. According to this database, this species is present in Austria with few occurrences. It is present in Belarus, Belgium, Croatia, Denmark, Italy (including Sardinia and Sicilia), Lithuania, Moldova, North Macedonia, Poland, Romania, Serbia, Slovenia, Turkey, Ukraine, and Azerbaijan, where *L. dispar* has been classified in the A1 list (pests are not present in the EPPO region) since 2007, and in Russia, which has been classified in the A2 (the presence of pests occurs locally in the EPPO region) since 2014. In Spain, there are reports from the XIX century. It is known as “lagarta peluda” in Spain, as “eruga peluda del suro” in Catalonia, and “eruga peluda” in the Balearic Islands. Most of the information available in Spain comes from old pest control books and technical leaflets published by some Spanish Regional Plant Health Services [57,58,66,67].

The dispersal of *L. dispar* is impressive since its females are not able to fly [12,57,58,59,124,125]. However, the spread of this species in new areas can occur through larvae that can disperse over short distances, as well as through the human-mediated movement, which can help spread some of the life-stages of this pest to over long distances [15,94]. Concretely, dispersal by larvae is accomplished by either their crawling from tree to tree or by larval ballooning, that is, a wind-borne movement, and is therefore limited to short distances (<100 m) [126,127,128,129]. Early instar larvae move to tree crowns, where they hang from strands of silk until the wind carries them locally to other trees, particularly in an urban situation [130]. In some cases, larvae may “balloon” several times before they start feeding [131]. However, it has been reported that the newly hatched larvae have been blown to distances of 56 km [61]. In addition, the primary pathway for the long-distance of *L. dispar* is human assisted (i.e., recreational travel; transportation of egg masses on firewood, household goods, and vehicles) [29,125,132,133]. For example, *L. dispar* has spread at a rate of 3 to 29 km per year since its introduction to the USA [134,135], which is based on a dispersal rate of more than 20 km per year for the period between 1966 and 1990 [131]. On the other hand, in the absence of anthropogenic movement, the natural spread that occurs through early instar-larvae ballooning could be as slow as 2.5 km/year [131].

The establishment of *L. dispar* in a new area depends on the temperature. For instance, in warm climates such as southern Florida, this species is not able to complete a full life cycle because there are not suitably low temperatures to complete the diapause [136]. In addition, in areas where the high summer temperatures exceed the optimum temperatures for larval and pupal development, the probabilities of establishing of this species are low [137,138]. Another important parameter for successful establishment in small, isolated populations or those at low density near range edges is the ability of individuals to successfully locate mates [139,140,141]. More specifically, the failure to establish *L. dispar* infestations ahead of the invasion is because of the unsuccessful detection of females by the males in low-density populations [139].

### 3.2. Outbreaks

*Lymantria dispar* populations appear to exist in one of the following four phases: innocuous, release, outbreak, and decline [94]. The innocuous phase is characterized by very low population levels. The release phase usually takes place over the course of one or two years and can result in population density increases of several orders of magnitude. During the outbreak phase, populations are high enough to cause noticeable defoliation to host trees. After this point, the decline phase follows, which is characterized by high levels of *L. dispar* mortality usually due to starvation or disease and the population crashes. Outbreaks in the whole area can last up to 10 years, but generally population densities in localized areas remain high for two or three years [142]. Usually, outbreaks of forest defoliators emerge in a synchronous manner at large spatial scales [143]. In contrast, *L. dispar asiatica*, which is found in Russia [144], exhibits asynchronous population outbreaks. This is because temperature varies considerably in Russia during winter (<−30 °C) vs. other European countries [145]. For several decades, the cyclic population dynamics and their genetic consequences have been an area of interest [146]. *Lymantria dispar* exhibits cyclic population dynamics [98,147,148]. More specifically, in Europe, which is the native territory of this pest, it exhibits cyclical outbreaks every 8–13 years [148]. For instance, in Spain, it is reported that cyclic outbreaks may last for a couple of years, followed by 7–11 years of non-outbreak period [58,66]. On the other hand, in North America, where the *L. dispar* is an invader species, it exhibits varying periodicity, every 4–5 years or 8–10 years, depending on forest type [33]. Severe outbreaks of *L. dispar* larvae can completely defoliate forest canopies, which have many short-term effects. These effects are related to the reduction in productivity or the reduction of seed crops, the increase in light to the forest floor, reduced transpiration that leads to increased water drainage from the forest, and a pulse of nitrogen and labile carbon to the forest floor [6,149,150]. In addition, as the host trees and seedlings can be completely defoliated and killed during outbreaks, this can cause changes in the composition of natural forests [151,152,153]. After an outbreak, *L. dispar* populations collapse due to viral or fungal disease, parasitism, predation, starvation, or adverse climatic conditions [154]. Apart from the environmental damage caused by the outbreaks of this pest, there are considerable costs in terms of economic losses and the cost of control measures [121,155,156].

*Lymantria dispar* infestations have positive and negative effects on wildlife. On the one hand, defoliation can lead to increased growth of shrubs, grasses, and herbs, providing an additional habitat for some wildlife species. On the other hand, defoliation may reduce or compromise the habitat for some wildlife species. For instance, bird eggs become vulnerable to predation due to reduced protection following defoliation of the tree leaves. Waterways can also be affected by outbreaks of *L. dispar*. Loss of canopy cover due to defoliation may increase the temperature of streams, which may be detrimental to aquatic organisms [157].

The most numerous and severe outbreaks have occurred in the Balkan peninsula because of the abundance of oak species and the climate (i.e., high temperatures and humidity deficits), which seems to be the optimum for the development and survival of *L. dispar* [98]. Indeed, in an earlier study, Schwenke [158] reported that the climate and the affinity of *L. dispar* for warmth and drought are causing an outbreak in southeastern and southern Europe. However, periodic outbreaks have been recorded since 1600 in Europe [159]. For example, outbreaks were reported in 1600 in Spain, 1750 in Germany, 1840 in Hungary, and 1880 in France [98]. In Serbia, 16 outbreaks were reported between 1862 and 1998, with the largest occurring in 1997 when 500,000 ha were infested [160]. Notably, in the mid-1950s and 1960s, outbreaks of this species were widespread and devastating in Europe, causing 70% defoliation in the Yugoslavian hardwood forests in 1957 [161]. However, the most severe epidemic in Europe took place between 1991 and 1995, affecting seven countries and hundreds of thousands hectares of forests, making control measures imperative [118,162]. During that period, France suffered the most, since this outbreak lasted longer there than in other Central European countries [118], where only in Alsace, an area of 23,500 ha, was defoliated in 1994 [163]. In Austria, 1500 ha were infested by the pest in 1993, causing severe defoliation on 400 ha of oak stands [164], whereas one year earlier in Switzerland, 2000 ha composed mainly of chestnut trees was defoliated. However, after the collapse of the *L. dispar* population in 1993, the trees recovered without any treatment [165]. An outbreak in Germany affected almost 2000 ha in 1992, while one year later, the infestation covered 47,000 ha, and in 1994, before the natural population collapsed, the pest was present in about 80,000 ha [118]. Furthermore, during this period, small outbreaks occurred in England, where the climate is rather unfavorable for *L. dispar* [118,166]. In Hungary, 22 outbreaks have been reported between 1843 and 2007, with the maximum damage (i.e., 212 thousand hectares) recorded in 2005 [98].

*Lymantria dispar* in Greece is considered one of the most important pests of oak trees. Greece has experienced several population outbreaks [167]. More recently, over the years 2016 and 2017, repeated outbreaks have been reported in different parts of Greece (i.e., Macedonia, Ipirus, and Thessaly) on evergreen pastures, where *Q. coccifera* predominates [130]. In Catalonia (Spain), during the last century, several outbreaks with massive defoliation were recorded, especially on *Q. suber* from the coastal and pre-coastal mountains of the Transversal Mountain System [168]. After a long period without important damage, new outbreaks were recorded in 2018–2020 in several counties, especially in the natural park of Montnegre-Corredor, in Barcelona province. Soldevila [169] and Stefanescu et al. [59] performed a detailed study in some affected forest of the Montnegre mountains of Catalonia (Barcelona province) where they determined the outbreak effects and the role of natural enemies as biocontrol agents. Defoliation of oak forest by *L. dispar* has been especially important in the Montnegre-Corredor park on *Q. rubra* and *Q. ilex*, after more than two decades without records of massive defoliations [65]. In that area in 2019, the damage covered nearly 15,000 ha [170] and it caused social alarm and concern in the forest owners, as more than 90% of the forest area of Catalonia is private. The attack not only affected *Quercus* sp., but also many specimens of *A. unedo*, *P. halepensis*, *Celtis* sp., *Castanea* sp., and other tree species.

During 2006–2008, the territory of the Russian Far East suffered from a *L. dispar* outbreak, which was characterized as the most severe since the 1930s [171]. In the Ugam-Chatkal range of Uzbekistan, 500 ha of various forest and fruit tree species were heavily damaged by *L. dispar* [172]. Furthermore, *L. dispar* outbreaks were reported in Northern Kazakhstan in 2017, damaging 247 ha of forest plants [173]. In North America, the history of outbreaks is related to the dispersal of *L. dispar* in new areas, where the preferred plant species predominate, followed by the rapid spread of populations due to the absence of a natural enemy complex. After a massive outbreak that occurred in 1979–1982, in 1981, at the peak of the outbreak in the USA, *L. dispar* defoliated more than 6 million ha of forests [174]. Later, the next outbreak occurred from 1989 to 1993, affecting forest areas in 12 different states [98].

## 4. Control of *L. dispar*

*Lymantria dispar* is considered one of the most destructive invasive pests, ranking third among the most costly invader insect pests worldwide [153]. Defoliation by *L. dispar* larvae can cause tree biomass losses up to 70%, while cumulative effects can result from consecutive defoliations [175,176]. From an economic perspective, *L. dispar* can massively affect timberlands and agricultural products, with high yearly costs on quarantined products. This pest impacts the aesthetics of forests and property values in urban areas, imposing high costs (disinfestation, tree removal, and replacement); it also burdens health systems and may negatively affect tourism. For instance, in the USA, the economic impact of this species was estimated at about USD 250 million per year in 2011 [121], but with the continued spread of *L. dispar* in North America, the economic impact was increased to USD 3.2 billion per year in just a few years [155].

In years with moth outbreaks, cutaneous reactions in humans can reach epidemic proportions in communities located near infested trees, the contact with airborne setae being mainly responsible. It is, therefore, crucial to control and manage *L. dispar* to mitigate the possible ecological, economic, and social impacts. Due to the negative impact of *L. dispar* on the forests, several pest control programs have been conducted against this pest to reduce populations and damage during outbreaks [177,178] or to slow its spread from infested to uninfected areas [134,179,180,181]. The first tools to control *L. dispar* were crude. Early trapping devices were baited with live females and other control tools were oil-fueled flamethrowers to destroy the life stages and microhabitats of the pest, as well as arsenic-based insecticides (copper acetoarsenite and lead arsenate) [52]. Current control measures include chemical and mechanical methods, natural predators, and silvicultural practices. In addition, the economic damage threshold of 100 egg clusters per 40 trees, which are equivalent to an average number of 2.5 egg clusters/tree, is used to make decisions about the initiation of management tactics [182,183,184].

The use of conventional insecticides, such as organophosphates, carbamates, and pyrethroids, is common in pest control, especially to prevent the spread of destructive insect species such as *L. dispar*, but this approach can be harmful to human health and the environment [185,186,187]. Although there is an increasing demand for alternative eco-friendly methods to control *L. dispar* (e.g., viruses, parasites, pheromones, fungi, or bacteria), chemical control is still the most common and effective method [188,189,190,191]. Starting from the 1980s, broad-spectrum insecticides used for *L. dispar* control have been replaced with bioinsecticides based on entomopathogenic viruses, bacteria, and fungi, as well as technologies such as mating disruption using sex pheromone traps [192,193].

### 4.1. Biological Control

#### 4.1.1. Natural Enemies

The infestation levels that *L. dispar* can cause in North America are much higher than those in Europe, due to more suitable biotic and abiotic conditions in the newly invaded areas [98]. Apparently, this phenomenon is partially due to the fact that its natural enemy complex in Europe is much more diverse than that in the Nearctic region, so that the natural regulation (i.e., naturally occurring biological control) of *L. dispar* populations is more efficient in Europe than in North America. The natural enemies include various predators, such as small mammals and birds. However, small mammals and birds do not come in numbers able to control large populations because the pest reproduces much faster than the predators. Pathogenic fungi require specific conditions to germinate and infect *L. dispar* larvae (high humidity, rainfall), making their effectiveness in a given year subject to local weather conditions. The natural enemies of *L. dispar* are presented in Table 2.

When the presence of *L. dispar* in an oak forest is at a high density, it becomes part of the food chain and can be an important food source for birds and small mammals (e.g., rodents) [194,204]. For instance, *P. leucopus*, which feeds on *L. dispar* larvae and pupae, can regulate low-density populations of this pest [194]. Moreover, mice can cause high mortality, for example, they killed 98% of deployed *L. dispar* pupae within 72 h in Ukraine [98] and caused more than 45% mortality in an artificial population of *L. dispar* pupae in Austria [205]. However, the abundance of small mammals and predation rates are affected by forest types and altitudes [206,207].

Recenlty, Soldevila [169] and Stefanescu et al. [59] considered the role of natural enemies in their studies in Catalonia. The authors identified *A. sylvaticus* and *C. russula* as predators with the capacity to maintain populations of *L. dispar* at low densities. As the abundance of small rodents is linked to the abundance of acorns, they suggested that after several years of low abundance of the fruits, the populations of rodents would decrease, allowing an increase in *L. dispar* populations. 

In earlier studies, birds had been proven to be one of the most important predators [194,208,209,210,211]. For instance, bird predation damaged 77% of the egg masses in Slovakia [208]. The predation rates of egg masses by birds in North America are between 65% and 89% [98,212]. Soldevila [169] and Stefanescu et al. [59] also reported the potential role of *P. major*, *G. glandarius*, *D. kizuki*, and *S. eiuropaea*.

*Calosoma sycophanta* is a predatory beetle that is known to occur in high densities during outbreaks of *L. dispar* [56,195,196,197,198,199]. Adults and larvae of this species are the main predator of larvae and pupae of *L. dispar* [98,213,214]. It is distributed in the Western Palearctic, covering the whole of Europe, northwest Africa, and Western and Central Asia [200,201,202,203]. It was imported from Europe to the USA as a natural enemy of *L. dispar* in 1906 and is now a well-established predator [214,215,216,217]. It has been reported that a pair of *C. sycophanta* adults and their offspring can predate more than 6000 *L. dispar* larvae and pupae within one season [198].

Stefanescu et al. [59] and Soldevila [169] reported the occurrence of *C. sycophanta* in Catalonia, confirming its capacity of this prey upon the larvae and pupae, as also reported by Weseloh [196]. *Calosoma sycophanta* was able to predate a large number of *L. dispar* when the density was high, but its role was much less important when *L. dispar* density was low. The above-named studies also stated that the parasitoids of the genera *Anastasus*, *Cotesia*, *Exorista,* and *Brachymeria* were present in the study area. However, vertebrates are more likely to cause high mortality than invertebrates [211]. In Slovakia, for instance, invertebrates caused 38% of egg mass predation, while vertebrates caused 62% [211]. In general, it has been suggested that predation by small mammals is able to keep *L. dispar* populations at low densities [205,206]. However, outbreaks occur at intervals, presumably due to human transfer of life stages of *L. dispar* from an infested to an uninfested area [132].

In the USA, since the early 20th century, numerous attempts have been carried out to eradicate or suppress the dispersal of *L. dispar*, but none have prevented the continued spread of the pest to the south and west of the country. Among the attempts was the development of a biological control program. The guiding philosophy for biological control in North America was to establish there all the natural enemies that attack *L. dispar* in Europe [218]. Thus, such a program mainly involved the introduction, rearing, and release of *L. dispar* enemies from Europe [198,218]. At least 20 parasitoids and predators have become established, but they have still been unable to control the moth populations [127]. By 1933, nine natural enemies of *L. dispar* had become established in North America. One of these was *C. sycophanta*, as well as some small hymenopterous egg parasitoids and some tachinid flies that attack the large larvae of *L. dispar* [219]. In addition to the other natural enemies in Europe, there are a large number of parasitoids that are able to attack *L. dispar*; of these, 109 species belong to the order Hymenoptera and 56 species belong to the order Diptera [63]. Despite this diversity of parasitoids, efforts have been conducted to establish parasitoids in the invasive range of *L. dispar*, but only a few parasitoid species can be considered as established there [50,83,220,221].

#### 4.1.2. Bioinsecticides or Pathogens

Among the control methods developed against *L. dispar* [222], the application of bioinsecticides has proven to be a successful approach for the control of this pest, with a low environmental impact [36,223]. Aerial applications of formulations based on the entomopathogenic bacterium *Bacillus thuringiensis* Berliner serovar *kurstaki* (*Btk*) exploit the highly specific mode of action of bacterial toxins that selectively target moth larvae [224]. This method is currently the most frequently used and effective to suppress *L. dispar* outbreaks since it has few biological and practical limitations [225]. However, there is some evidence of side effects on non-target Lepidoptera that live in the forest ecosystem [226].

Several factors should be taken into account to increase the effectiveness of *Btk* applications against *L. dispar* [227], including the timing of application relative to the phenology of second instar larvae [228,229], dose [180,230], the size of droplet, and density on foliage of insecticides based on *Btk* [231].

Stefanescu et al. [59] evaluated the defoliation that occurred in plots of a damaged area in 2020 on trees that had been already attacked or not in 2019. The cumulated defoliation that occurred in 2020 (2019 + 2020 damage) was estimated at 20% to 60%. As a measure to reduce damage, the Plant Health Services of the Department of Agriculture of the Catalonia Government planned a program based on the treatment of the most affected forests with *Btk,* and around 2500 ha were sprayed in order to maintain the productivity of the forest, especially areas of *Q. suber* exploited to obtain cork. The cost of spraying was estimated at 50 EUR/ha. The efficacy of such treatments was low and did not have any significant effect on the reduction of defoliation [59], and the use of *Btk* spraying for controlling damage of *L. dispar* was seriously questioned. Several reasons were given: (1) the development of the larval population in one locality is not synchronous and there are several larval instars the at the same time; (2) the negative effect that the treatment may have on the non-target species: although *Btk* is a selective bioinsecticide widely used in integrated pest management, it negatively impacts lepidopteran larvae and some predator species such as the green lacewing *Chrysoperla carnea* (Stephens) (Neuroptera: Chrysopidae) [59]. Indirectly, the reduction of lepidopteran larvae in sprayed *Btk* areas can lead to a reduction in the natural enemies that feed on them. This last aspect of loss of biodiversity is more relevant in protected areas such as the Montnegre of Catalonia. Although the study of Stefanescu et al. [59] did not measure and compare the productivity of sprayed and unsprayed forest, they argue that *Q. suber* forest has a notable resilience to *L. dispar* outbreaks.

During the outbreak of the 1990s in central Europe, control measures against *L. dispar* in France (1995 epidemic) were carried out in an area of 2500 ha using *Btk*. In Slovakia and the Czech Republic in the 1993–1994 outbreaks in oak forests, treatments with *Btk* were necessary. In Germany, in the outbreaks of 1993 and in 1994, *Btk* was applied by helicopter to infested areas. However, mainly due to adverse weather conditions, this treatment failed to provide sufficient protection on the affected forest stands [118]. More recently in Greece, the field treatments with *Btk* against *L. dispar* showed that this biopesticide caused 66% mortality to the second instar larvae after 4–5 days [36].

*Lymantria dispar* has been treated with other biocontrol agents such as entomopathogenic fungi and entomopathogenic nematodes [36,38,39,40,41,42,43]. The entomopathogenic fungus *Entomophaga maimaiga* Humber, Shimazu and Soper 1988 (Entomophthoromycota: Entomophthorales) was introduced to the United States from Japan for the biological control of *L. dispar* in the early 1900s; however, its introduction was unsuccessful [232]. Much later, in 1989, there was an unexpectedly high percentage of dead larvae due to this fungus [233,234], probably because it had been accidentally introduced to North America after 1971 [235]. In late 1990s, *E. maimaiga* was introduced to Bulgaria from North America [236]. Since then, it rapidly spread across Europe, indicating that this continent is suitable for the survival and development of the fungus [237]. Field studies in Europe documented that *L. dispar* larvae exhibited 98% mortality when infected by *E. maimaiga* [238,239,240], suggesting the high potential of this fungus to become an important management tool [237]. The entomopathogenic nematode *Steinernema carpocapsae* (Weiser, 1955) (Rhabditida: Steinernematidae), which was used for the first time in Greece in field trials against this pest, killed 69% of the second instar larvae, indicating that it has a great potential as a control agent [36].

Another group of entomopathogens is the baculoviruses that are very specific microorganisms [241] that cause fatal infections to larvae after the ingestion of viral particles. The bioinsecticidal activity is associated with crystalline occlusion bodies that, after their ingestion by susceptible insects, release occlusion-derived viruses (ODVs) that infect the host midgut epithelial cells. The production of a second type of virions, namely, budded viruses (BVs), responsible for the infection spread in the host body [242]. The *Lymantria dispar* multiple nucleopolyhedrovirus (LdMNPV) is very specific to this pest and is therefore environmentally safe. It was apparently introduced accidentally with *L. dispar* in North America [243]. This virus naturally causes epizootics, resulting in the decline of the pest population after two or three years of outbreaks [244]. For instance, Kurenshchikov et al. [171] reported that LdMNPV suspended the population of *L. dispar* after the outbreak in Khabarovsk Krai (Far East region of Russia) in 2006–2008. However, it can be produced in large quantities only by infecting live *L. dispar* larvae. Thus, it is produced in limited quantities, sufficient only to treat <550 km^2^ per year [125]. At first, virus production was costly since efficient host larval breeding techniques were limited [245]. However, research efforts have revealed that virus yields can be increased by using late-instar (i.e., fifth instar) female larvae where, in combination with new larval processing methods (i.e., using freeze-dried, intact larvae), the cost of the production of the virus can be reduced effectively [245]. The USDA Animal and Plant Health Inspection Service and the Forest Service have investigated practical ways to use the virus as biological insecticide. Thus, it was registered with the Environmental Protection Agency in 1978 as Gypchek [243]. Lewis and Yendol [246] reported that Gypchek adequately controlled *L. dispar* in forest trials. In addition, in North America, the virus is considered to be the most significant factor causing the collapse of *L. dispar* populations [98]. More recently, Ruiu et al. [247] suggested a multi-year integrated program that would include the combined use of *Btk* to reduce infestations and nucleopolyhedrovirus in order to regulate population dynamics. The disease is often referred to as “wilt” because of the soft, limp appearance of diseased larvae [11].

*Lymantria dispar* can host several microsporidian pathogens, some of which have been considered for inoculative releases in North America [248]. *Vairimorpha disparis* (Microsporidia, Burellenidae) and *Nosema lymantriae* (Microsporidia, Nosematidae) infect *L. dispar* larvae when the spores are ingested on food. The developmental cycle begins in the midgut and eventually leads to the formation of primary spores [217]. *Vairimorpha disparis* is a parasite of the fat body. The environmental spores are transmitted between host larvae and can be found in the fat body after 7 days. After 10 days, the fat body is full of spores, and under laboratory conditions, an infected larva dies on average 4 weeks after infection [249]. Horizontal transmission takes place when spores are released from the decomposing cadaver. *Nosema lymantriae* is a systemic parasite that affects the silk glands, the fat body, the gonads, and the Malpighian tubules of a host larva. Most of the infected larvae will die 4 weeks after infection. Horizontal transmission of this species begins after the end of the latent period when the spores are released with feces about two weeks after infection, and also continues after the death of the host larva when its cadaver decomposes [249]. Furthermore, infected females can transmit *Nosema lymantriae* transovarially to their offspring [250].

In Central Europe, it has been reported that pathogens can cause higher mortality than parasitoids [208,251,252]. Since entomopathogens can act against *L. dispar*, Stephanescu et al. [59] reported that it is probable that the baculovirus LdMNPV is present in Catalonia, although there are no records of this. In conclusion, it seems that natural enemies play a role in maintaining *L. dispar* populations under outbreak thresholds or in reducing population levels after outbreaks.

### 4.2. Traps and Attractants

The development of the sex pheromone *cis*-7,8-epoxy-2-methyloctadecane (disparlure) that attracts *L. dispar* males took place in the 1970s [253,254]. The existence of the pheromone was known in the early 1900s, and the tips of the female abdomen were extracted into organic solvents to provide pheromone that could be used in trapping research as early as 1932 [255]. This pheromone has been used extensively for the detection of this pest [134] and is able to attract all of the different subspecies of *L. dispar* [12]. The commercially available pheromone disparlure provides an extremely sensitive and host-specific research tool that is effective even when moths occur at very low population densities [6,256,257,258]. Without such a sensitive trapping tool, it would be extremely difficult to engage in management efforts that depend on early detection [125]. The detection of low-density populations of this pest is essential for eradication campaigns. Moreover, pheromone-baited traps are effective at both low and high densities, and this method is considered the most sensitive method for detecting *L. dispar* [15].

The sexual behavior of males [244,259], as well as the physical characteristics of a trap, such as the shape, size, color, and the location of entry ports, must be taken into account when designing a trap [6]. There is a wide variety of pheromone trap designs from disposable cardboard traps with a sticky inner surface to various canister-type or “milk carton” traps made of cardboard or reusable plastic that contain an insecticide to kill the captured moths [11]. Strategies to slow the spread of *L. dispar* are based on pheromone traps for detecting and delimiting small, isolated populations immediately after establishment [193,260]. These infestations can then be targeted for mass trapping, mating disruption, application of microbial insecticides, or other appropriate methods to limit the population growth and spread of the pest [261,262,263].

Mating disruption (MD) has long been regarded as a viable option for non-chemical control, especially in the case of Lepidoptera [264]. For MD, artificial male pheromone is released in a treated area in order to compete with pheromone produced by calling females, and this can drastically reduce mate finding by males and thus mating [265]. Currently, this method is used to control a large number of insect pest species, mainly lepidopteran species, but not exclusively so [264,265,266,267,268,269,270,271,272]. MD has been successfully marketed for a very wide range of moth species of economic importance [273,274,275].

With this method, mating may be delayed, which contributes to reduced fecundity [276,277] and egg fertilization [277]. Moreover, MD can reduce the risk of insecticide resistance because mating among resistant individuals is decreased [278,279,280]. Since the target organism is not killed by this method, non-targeted effects are believed to be rare [179]. It is also compatible with other pest management strategies, such as biological control [261]. The discovery of disparlure also helped to develop and optimize host-specific MD tactics that are also considered a very useful control tool [193,254,269].

MD has been proven to be effective against low-density populations of *L. dispar* in the USA [179,281,282,283,284]. On the other hand, it may not work in high-density populations where female moths are abundant and easy for males to locate, making it ineffective without the combined use of other control measures. As a univoltine species, *L. dispar* is ideal for MD, as a single annual application may lead to rapid population suppression, without the need to reapply the method; however, the timing of the application is critical, i.e., before males mate with females. Although *L. dispar* is a serious threat for both the environment and human health, the MD method has never been evaluated on a large scale in Europe as a reliable management tool.

An alternative control method is the use of trunk barriers, which can take advantage of the dispersal behavior of the larvae [80]. The use of burlap bands wrapped around the trunk is an old technique that helped to collect and destroy the larvae [52]. In the early 1900s, the protection of trees from *L. dispar* in Massachusetts was based on the extensive use of sticky barrier bands [285]. Later, burlap bands and similar devices were used to detect and monitor *L. dispar* populations [84,286,287]. For instance, burlap bands that were placed around the base of the trunk have been used to trap and monitor late-instar larvae; the larvae use the burlap bands as a preferred resting site during the day [288]. Moreover, the first-instar larvae that move upward on the tree stem after hatching can be trapped in the sticky barrier bands [289,290].

### 4.3. Chemical and Biorational Control

There are only a few insecticides registered for *L. dispar* control in Europe, such as insect growth regulators (IGRs), which are biorational products, and Spinosad, a naturally derived product [36,37]. Other insecticides may be used to effectively control *L. dispar*, but since their potential harm to other species is considered too high, they are not recommended for widespread use. In the past, the application of pesticides to prevent *L. dispar* attack has been shown to have a greater effect on the bird populations than on *L. dispar* themselves [291]. Moreover, since this species may occur in urban/recreational areas, chemical control is even more challenging as many of the registered pesticides cannot be used due to risks of exposure to mammals. Prior to 1966, area-wide eradication programs by spraying with dichloro diphenyl trichloro ethane (DDT) were considered effective. However, DDT and similar insecticides were eventually banned due to environmental concerns about their toxic effects becoming recognized, leading to research in finding alternatives to broad-spectrum synthetic pesticides [292].

In North America, massive aerial applications of pesticides have been made against *L. dispar* populations, aiming largely at suppressing their outbreaks [192]. For instance, the application of chemical pesticides (e.g., DDT) to millions of acres of forest in the United States took place annually during the 1940s and 1950s [293]. In the United States, the chemical insecticide diflubenzuron, which is a molting disruptor, was widely used during the 1980s [182] and in some European forests until recently [294]. For example, in Slovakia and the Czech Republic in the 1993–1994 outbreaks, the application of diflubenzuron in oak forests was used to control this pest. In Germany, the same treatment was applied to three-quarters of the infested area, which was proven to be more effective than treatment with *Btk* [118]. Although this insecticide is not toxic to vertebrates, concerns have been raised about its adverse effects on invertebrates [295] and the potential effects of the 4-chloroalinine metabolite on human health [296]. Later, tebufenozide, which is a molting hormone agonist, was approved as an alternative to diflubenzuron due to its specific action on Lepidoptera [178]. This compound now plays an important role in *L. dispar* management in the United States [89]. It is also the preferred option for some European countries (e.g., Germany) because of its reliable suppression of *L. dispar* populations [297].

In a recent study conducted in northern Greece, Papadopoulou et al. [36] reported that a product containing 24% metaflumizone had a rapid action (1–2 days) against second-instar larvae of *L. dispar*, causing 88% mortality, while an IGR containing 15% teflubenzuron was less effective, killing 76% of second instar larvae. In Spain, the authorized insecticides comprise azadiractin, cypermetrin, and indoxacarb [66]. Spraying with aircraft or some other flight system can only be performed by government Plant Health Services. The semi-synthetic bioinsecticide emamectin benzoate (EMB), which is derived from naturally occurring avermectin, is a widely used control agent for agricultural and forestry pests [298,299]. The determination of the sublethal concentrations of EMB and their effects on *L. dispar* was conducted by Xu et al. [300]. These authors found that sublethal concentrations of EMB can inhibit the growth of larvae, which is related to midgut damage, digestive dysfunction, and nutritional metabolism disorders. They also provided a theoretical basis for understanding the sublethal effect of EMB and its application to the prevention and control of *L. dispar*. According to Cannon et al. [166], an eradication program was applied in the UK, including three axes: (1) investigation for detecting egg clusters or larvae, and captures of adults using pheromone traps; (2) chemical treatments of larvae; and (3) extensive information of the public about the importance of the pest. This procedure resulted in a decrease in the captures of adult males in comparison with the captures of the first two years of the program.

In the past decade, increasing attention has been paid to the development of insecticides based on unmodified nucleic acid fragments, with an emphasis on antisense DNA fragments [301,302] and double-stranded RNA fragments [303,304]. These insecticides are considered to be the next-generation control agents that have numerous advantages over broad-spectrum products. They can combine the affordability and rapid action of chemical insecticides combined with the selectivity of biological preparations. The nucleic acid synthesis technologies are becoming less expensive, making DNA insecticides and RNA preparations increasingly economical, and their affordability can be compared to that of chemical insecticides. So far, antisense DNA-based insecticides are the only nucleic acid preparations being developed to control *L. dispar* [305].

Recently, Oberemok et al. [306] suggested a novel biotechnology for pest control, using a DNA insecticide that has improved insecticidal action based on a new antisense oligoRIBO-11 sequence from the 5.8S ribosomal RNA gene. This novel insecticide caused high mortality among *L. dispar* larvae reared in the laboratory and those collected from the forest. Furthermore, the insecticidal potential of three different 10–12 nucleotide long antisense sequences from the 5.8S ribosomal RNA gene of *L. dispar* against its larvae was compared. The results showed that antisense fragments of 10 and 11 nucleotides (oligoRIBO-10 and oligoRIBO-11) caused higher larval mortality than the 12 nucleotide long fragment (oligoRIBO-12) [307]. In addition, this oligoRIBO-11 insecticide was more affordable and acted faster than the previous preparations developed by Oberemok et al. [307], according to longer antisense fragments of anti-apoptosis genes of the baculovirus–host system.

### 4.4. Essential Oils

Many insecticides have been removed from the market due to the development of resistance and the severe environmental disturbances (i.e., persistence of residues and adverse effects on non-target organisms) [308,309,310,311,312,313]. Intensive work is being done to find alternative environmentally friendly pest control methods that will be effective [314,315,316,317,318,319]. In this context, plant-based products have been considered as potential control agents for pests, including *L. dispar* [320,321]. Recently, plant essential oils (EOs), which are complex mixtures of compounds that act as a defense agents against pests and pathogens and provide protection to the plant from heat and cold have attracted attention [322,323]. EOs are also generally recognized as safe biopesticidal agents [324,325,326,327,328].

Several EOs can be promising “green” alternatives to chemical insecticides for the control of *L. dispar,* as they have been examined under laboratory conditions. For instance, Moretti et al. [329] found a high digestive toxicity of rosemary, *Rosmarinus officinalis* L., and thyme, *Thymus herba-barona* Loisel. (Lamiales: Lamiaceae), EO emulsions to second and third instar larvae of *L. dispar* after 3 days of exposure. In addition, the EOs from basil, *Ocimum basilicum* L. (Lamiales: Lamiaceae); *Athamanta haynaldii* L. (Apiales: Apiaceae); and nutmeg, *Myristica fragrans* Houtt. (Magnoliales: Myristicaceae) had low to moderate residual contact and digestive toxicity, but they had good antifeedant activity against second instar larvae [3,330].

More recently, Devrnja et al. [331] examined the effect of the EO of the tansy, *Tanacetum vulgare* L. (syn. *Chrysanthemum vulgare* L.) (Asterales: Asteraceae), at three concentrations (i.e., 0.1, 0.5, and 1% *v*/*v*), on the survival and molting of second-instar larvae, as well as on the nutritional indices of the fourth-instar larvae of *L. dispar*. Exposure of the second instar larvae to tansy EO (residual contact toxicity) caused low mortality (<10%), but larval development was significantly extended, i.e., the proportion of larvae that molted into the third instar was decreased after 120 h of exposure in comparison with the control larvae (92% molted into the third instar). Consequently, delayed ecdysis, which is associated with prolonged growth, could extend the period for which larvae are exposed to their natural enemies. However, in a digestive toxicity assay in which tansy EO was incorporated into the diet, the highest concentration of the EO (i.e., 1% *v*/*v*) caused high mortality and a lack of molting after 120 h of consumption. Oxygenated monoterpenes were the predominant group of compounds with 93.5% in tansy EO [331,332]. Terpenes can act as deterrents or toxicants to *L. dispar* larvae [333], indicating that compounds from EOs could be isolated and used for the control of this species. However, the sensitivity of exposed larvae depends on the terpene structures and larval age [331].

Kostić et al. [321] evaluated the impact of different concentrations of essential oils (EOs) from the seeds of three Apiaceae plants, namely, anise, *Pimpinella anisum* L.; dill, *Anethum graveolens* L.; and fennel, *Foeniculum vulgare* Mill. (Apiales: Apiaceae), on the behavior, mortality, molting, and nutritional physiology of *L. dispar* larvae. The authors also compared EO efficacy with the commercial insecticide NeemAzal^®^-T/S (neem). Their results showed that the tested EOs may be a promising strategy for *L. dispar* control since they provided strong negative effects on survival and consumption in second-instar larvae and impairment of nutritional physiology in fourth-instar larvae. Anise EO was proven to be the best antifeedant, while dill EO caused the highest mortality. In addition, at the concentration of 0.5%, the three EOs performed better than the commercial insecticide neem in reducing relative growth rate, efficiency of conversion of ingested food, approximate digestibility, and efficiency of conversion of digested food of the fourth instar larvae of *L. dispar*. Although essential oils represent an eco-friendly management tool, their cost is high.

## 5. Public Health Concerns

Envenomation (i.e., infusion of an insect secretion into a human’s body) caused by moth or butterfly larvae in humans is known as “erucism” or “caterpillar dermatitis”, coming from the Latin “eruca”, which means caterpillar [334]. This kind of dermatitis has been noted since ancient Greek times [88,113,335]. Caterpillar envenomation constitutes a serious public health issue of international importance [336].

The setae of the caterpillars, which are filled with toxins (such as proteolytic enzymes, histamine, and other pro-inflammatory substances), are responsible for the allergic reactions they cause to humans or to animals [337,338,339,340]. The bristles are able to penetrate the subcutaneous tissue and release toxins [336,341], causing cutaneous reactions, such as immediate severe pain, erythema, and edema [336]. Of the three types of urticating hairs that exist in Lepidoptera, *L. dispar* larvae have modified setae [342]. The presence of histamine has been reported in the setae of *L. dispar* larvae [343,344,345,346,347]. In addition, histamine or histamine analogues have been isolated in other lepidopteran species of medical importance found in Europe, such as the pine-tree lappet moth, *Dendrolimus pini* (L.) (Lepidoptera, Lasiocampidae), and the browntail moth, *Euproctis chrysorrhoea* L. (Lepidoptera: Lymantriidae) [348]. Moreover, first instar larvae of *L. dispar* carry setae that are impregnated with nicotine, while this substance occurs in a lower concentration in last instar larvae [342,349].

It has long been recognized that *L. dispar* can cause dermatitis in humans [350], especially in laboratory staff [351]. In addition, the early instar larvae are considered more allergenic than mature larvae [130,352]. Several studies have shown that a large proportion of workers in breeding facilities of this species for experimental purposes developed pruritic urticaria on exposed skin after contact with its larvae [353,354]. Other symptoms associated with allergic reactions to larvae are eye irritation, rhinitis, and shortness of breath [354,355].

Very little information is available on the prevalence of cutaneous reactions focusing on children and in the general adult population. The first community-wide outbreak of *L. dispar* dermatitis was reported in the United States in 1981 [343]. That year, a massive outbreak of larvae was recorded in northeastern United States during spring [356], where people outdoors came in to contact with first instar larvae [352]. Thousands of people presented skin irritation, which was described as unusual pruritic dermatitis with stinging, while some people had respiratory difficulties [343,345]. Moreover, the skin lesions of the patients’ occurred within 12 h of contact with *L. dispar* larvae [343,345,352,357]. After the outbreak of 1981, there were no other reports of an allergic reaction to this pest [357] until the spring of 1990, when six new cases with clinical and histopathological features were described [88]. In addition to skin irritation, respiratory problems (e.g., rhinitis or shortness of breath and eye irritation) were reported [88,358].

Although larvae cause dermatitis with a pruritic eruption that lasts from 4 to 7 days, very few clinical and epidemiological studies have been conducted in this species [348,352,359]. Moreover, the etiology of *L. dispar* erucism and lepidopterism has not been fully clarified [115,357]. An epidemiologic study was carried out during the outbreak of *L. dispar* in 1981 that compared a severely infested and a lesser infested area [353]. The authors found that the highest risk factors for developing *L. dispar* dermatitis were previous history of hay fever, a history of a similar rash a year ago and direct physical contact or indirect exposure (e.g., hanging laundry outdoors) [352,353,355]. Furthermore, Beaucher and Farnham [345] conducted patch testing using the hairs of *L. dispar* larvae in 8 patients with a history of *L. dispar* dermatitis as well as in 11 persons without history that were used as controls. Generally, patch testing with moth or larvae setae revealed the presence of an immediate hypersensitivity, delayed-type hypersensitivity, or both [360]. In all patients with a history of dermatitis, patch testing caused delayed papulovesicular reactions, while only 1 out of 11 controls reacted, indicating a delayed-type hypersensitivity response [345]. Consequently, the mechanism of *L. dispar* erucism and lepidopterism probably involves local and pulmonary histamine release and delayed-type hypersensitivity reactions in susceptible persons [115,358]. In cases of *L. dispar* dermatitis, patients receive topical and parenteral antihistamines, oral or parenteral corticosteroids, and bronchodilators (as indicated for bronchospasm) [115].

## 6. Conclusions and Future Perspectives

*Lymantria dispar* is a pest of economic importance that can seriously disrupt forest ecosystems worldwide [361]. Considering the elevated environmental and economic impact of this species, a holistic approach is required rather than ad hoc interventions. In support of this, the European Directive 2009/128/CE of the European Parliament and of the Council of the European Union have established a framework for action so as to achieve sustainable use of pesticides, i.e., reducing their risks and impacts on human health and the environment. This framework promotes the use of integrated pest management (IPM) and the use of alternative non-chemical approaches or techniques [362]. Risks from exposure to chemicals in public parks, gardens, sports and recreational areas, school grounds, children’s playgrounds, or areas close to healthcare facilities are high and should therefore be minimized or prohibited. New approaches regarding monitoring and predicting outbreaks and controlling high-density populations should be introduced in forest and urban habitats. For example, novel trap types should be designed and tested extensively for the capture of larvae and adults. These trap devices can be incorporated in trap monitoring systems that will optimize trap captures at the very early beginning of the presence of the insect. Thus, effective management approaches should be applied accurately, such as the mating disruption method. For this purpose, the European Commission has initiated financial support to universities, research institutes, and private companies to work simultaneously in large-scale field experiments towards the development effective management methods against this serious pest.

## Figures and Tables

**Table 1:** Susceptible, preferred, and resistant tree species to *Lymantria dispar* larvae.

Common Name	Scientific Name	Order	Family	Categorization	Reference
European crab apple	*Malus sylvestris* (L.) Mill.	Rosales	Rosaceae	Susceptible	[17]
Bigtooth aspen	*Populus grandidentata* Michaux	Mapighiales	Salicaceae	Susceptible	[17]
Quaking aspen	*P. tremuloides* Michx.	Mapighiales	Salicaceae	Susceptible	[17]
Boxelder	*Acer negundo* L.	Sapindales	Sapindaceae	Susceptible	[17]
American mountain ash	*Sorbus americana* Marshall	Rosales	Rosaceae	Susceptible	[17]
Sweetgum	*Liquidambar styraciflua* L.	Saxifragales	Altingiaceae	Susceptible	[17]
Basswood	*Tilia* spp.	Malvales	Malvaceae	Susceptible	[17]
Birch	*Betula* spp.	Fagales	Betulaceae	Susceptible	[17]
Larch	*Larix* spp.	Pinales	Pinaceae	Susceptible	[17]
Oak	*Quercus* spp.	Fagales	Fagaceae	Susceptible	[17]
Willow	*Salix* spp.	Malpighiales	Salicaceae	Susceptible	[17]
Alder	*Alnus* spp.	Fagales	Betulaceae	Preferred	[10]
Hawthorn	*Crataegus* spp.	Rosales	Rosaceae	Preferred	[10]
Hazelnut	*Corylus* spp.	Fagales	Betulaceae	Preferred	[10]
Hornbeam	*Carpinus* sp.	Fagales	Betulaceae	Preferred	[10]
Serviceberry	*Amelanchier* spp.	Rosales	Rosaceae	Preferred	[10]
Sumac	*Rhus* spp.	Sapindales	Anacardiaceae	Preferred	[10]
Hemlock	*Tsuga canadensis* (L.) Carrière	Pinales	Pinaceae	Resistant	[10,17]
Yellow birch	*Betula alleghaniensis* Britt.	Fagales	Betulaceae	Resistant	[10,17]
Eastern white pine	*Pinus strobus* L.	Pinales	Pinaceae	Resistant	[10,17]

**Table 2:** Predators and parasitoids of *Lymantria dispar* larvae.

Predators			
Species Name	Order	Family	Reference
*Peromyscus leucopus* (Rafinesque, 1818)	Rodentia	Cricetidae	[194]
*Apodemus sylvaticus* (Linnaeus, 1758)	Rodentia	Muridae	[59,169]
*Crocidura russula* (Hermann, 1780)	Eulipotyphla	Soricidae	[59,169]
*Parus major* Linnaeus, 1758	Passeriformes	Paridae	[59,169]
*Garrulus glandarius* (Linnaeus, 1758)	Passeriformes	Corvidae	[59,169]
*Dendrocopos kizuki* (Temminck, 1836)	Passeriformes	Picidae	[59,169]
*Sitta eiuropaea* Linnaeus, 1758	Passeriformes	Sittidae	[59,169]
*Calosoma sycophanta* L.	Coleoptera	Carabidae	[56,59,169,195,196,197,198,199,200,201,202,203]
**Parasitoids**			
*Acropimpla didyma* (Gravenhorst)	Hymenoptera	Ichneumonidae	[44]
*Aleiodes pallidator* Thunberg	Hymenoptera	Braconidae	[44]
*Anastatus bifasciatus* (Geoffroy)	Hymenoptera	Eupelmidae	[44]
*Anastatus catalonicus* Bolivar & Pieltain	Hymenoptera	Eupelmidae	[44]
*Anastatus japonicus* Ashmead	Hymenoptera	Eupelmidae	[44]
*Apanteles impurus* (Nees)	Hymenoptera	Braconidae	[44]
*Apanteles lacteicolor* Viereck	Hymenoptera	Braconidae	[44]
*Apanteles xanthostigma* (Haliday)	Hymenoptera	Braconidae	[44]
*Apechthis capulifera* (Kriechbaumer)	Hymenoptera	Ichneumonidae	[44]
*Apechthis compunctor* (L.)	Hymenoptera	Ichneumonidae	[44]
*Apechthis quadridentata* (Thomson)	Hymenoptera	Ichneumonidae	[44]
*Apechthis rufata* (Gmelin)	Hymenoptera	Ichneumonidae	[44]
*Aphantorhaphopsis samarensis* (Villeneuve)	Diptera	Tachinidae	[44]
*Banchus falcatorius* (Fabricius)	Hymenoptera	Ichneumonida	[44]
*Barylypa pallida* (Gravenhorst)	Hymenoptera	Ichneumonida	[44]
*Baryscapus oophagus* (Otten)	Hymenoptera	Eulophidae	[44]
*Blepharipa pratensis* (Meigen)	Diptera	Tachinidae	[44]
*Blepharipa schineri* (Mesnil)	Diptera	Tachinidae	[44]
*Blondelia nigripes* (Fallén)	Diptera	Tachinidae	[44]
*Blondelia piniariae* (Hartig)	Diptera	Tachinidae	[44]
*Bothriothorax altensteinii* Ratzeburg	Hymenoptera	Encyrtidae	[44]
*Bothriothorax paradoxus* Dalman	Hymenoptera	Encyrtidae	[44]
*Brachymeria inermis* (Fonscolombe)	Hymenoptera	Chalcididae	[44]
*Brachymeria minuta* (L.)	Hymenoptera	Chalcididae	[44]
*Brachymeria secundaria* (Ruschka)	Hymenoptera	Chalcididae	[44]
*Brachymeria tibialis* Walker	Hymenoptera	Chalcididae	[44]
*Campoplex difformis* (Gmelin)	Hymenoptera	Ichneumonidae	[44]
*Carcelia gnava* (Meigen)	Diptera	Tachinidae	[44]
*Casinaria tenuiventris* (Gravenhorst)	Hymenoptera	Ichneumonidae	[44]
*Chouioia cunea* Yang	Hymenoptera	Eulophidae	[44]
*Cirrospilus pictus* Nees	Hymenoptera	Eulophidae	[44]
*Compsilura concinnata* (Meigen)	Diptera	Tachinidae	[44]
*Cotesia gastropachae* (Bouché)	Hymenoptera	Braconidae	[44]
*Cotesia glomerata* (L.)	Hymenoptera	Braconidae	[44]
*Cotesia melanoscela* (Ratzeburg)	Hymenoptera	Braconidae	[44]
*Cotesia melitaearum* (Wilkinson)	Hymenoptera	Braconidae	[44]
*Cotesia neustriae* (Tobias)	Hymenoptera	Braconidae	[44]
*Cotesia ocneriae* (Ivanov)	Hymenoptera	Braconidae	[44]
*Cotesia praepotens* (Haliday)	Hymenoptera	Braconidae	[44]
*Cotesia rubripes* (Haliday)	Hymenoptera	Braconidae	[44]
*Cotesia spuria* (Wesmael)	Hymenoptera	Braconidae	[44]
*Deuterixys carbonaria* (Wesmael)	Hymenoptera	Braconidae	[44]
*Doryctes leucogaster* (Nees)	Hymenoptera	Braconidae	[44]
*Drino gilva* (Hartig)	Diptera	Tachinidae	[44]
*Drino inconspicua* (Meigen)	Diptera	Tachinidae	[44]
*Dusona blanda* (Förster)	Hymenoptera	Ichneumonidae	[44]
*Elachertus charondas* Walker	Hymenoptera	Eulophidae	[44]
*Elasmus nudus* Nees	Hymenoptera	Eulophidae	[44]
*Euceros serricornis* (Haliday)	Hymenoptera	Ichneumonidae	[44]
*Euceros superbus* Kriechbaumer	Hymenoptera	Ichneumonidae	[44]
*Eulophus cyanescens* Bouček	Hymenoptera	Eulophidae	[44]
*Eulophus larvarum* L.	Hymenoptera	Eulophidae	[44]
*Eulophus slovacus* Bouček	Hymenoptera	Eulophidae	[44]
*Eupelmus annulatus* Nees	Hymenoptera	Eulophidae	[44]
*Eupelmus urozonus* Dalman	Hymenoptera	Eulophidae	[44]
*Euplectrus liparidis* Ferrière	Hymenoptera	Eulophidae	[44]
*Eurytoma appendigaster* Swederus	Hymenoptera	Eurytomidae	[44]
*Eurytoma goidanichi* Bouček	Hymenoptera	Eurytomidae	[44]
*Eurytoma verticillata* (Fabricius)	Hymenoptera	Eurytomidae	[44]
*Exeristes roborator* (Fabricius)	Hymenoptera	Ichneumonidae	[44]
*Exorista amoena* (Mesnil)	Diptera	Tachinidae	[44]
*Exorista larvarum* (L.)	Diptera	Tachinidae	[44]
*Exorista segregata* (Rondani)	Diptera	Tachinidae	[44]
*Gelis agilis* (Fabricius)	Hymenoptera	Ichneumonidae	[44]
*Gelis areator* (Panzer)	Hymenoptera	Ichneumonidae	[44]
*Glyptapanteles porthetriae* (Muesebeck)	Hymenoptera	Braconidae	[44]
*Glyptapanteles vitripennis* (Curtis)	Hymenoptera	Braconidae	[44]
*Gregopimpla inquisitor* (Scopoli)	Hymenoptera	Ichneumonidae	[44]
*Gryon howardi* (Mokrzecki and Oglobin)	Hymenoptera	Scelionidae	[44]
*Gryon hungaricum* (Szabó)	Hymenoptera	Scelionidae	[44]
*Gryon lymantriae* (Masner)	Hymenoptera	Scelionidae	[44]
*Hemiteles pulchellus* Gravenhorst	Hymenoptera	Ichneumonidae	[44]
*Hyposoter tricoloripes* (Viereck)	Hymenoptera	Ichneumonidae	[44]
*Ichneumon sarcitorius* L.	Hymenoptera	Ichneumonidae	[44]
*Iseropus stercorator* (Fabricius)	Hymenoptera	Ichneumonidae	[44]
*Itoplectis alternans* (Gravenhorst)	Hymenoptera	Ichneumonidae	[44]
*Itoplectis enslini* (Ulbricht)	Hymenoptera	Ichneumonidae	[44]
*Itoplectis kolthoffi* (Aurivillius)	Hymenoptera	Ichneumonidae	[44]
*Itoplectis viduata* (Gravenhorst)	Hymenoptera	Ichneumonidae	[44]
*Lymantrichneumon disparis* (Poda)	Hymenoptera	Ichneumonidae	[44]
*Lysibia nana* (Gravenhorst)	Hymenoptera	Ichneumonidae	[44]
*Melittobia acasta* Walker	Hymenoptera	Eulophidae	[44]
*Mesochorus confusus* Holmgren	Hymenoptera	Ichneumonidae	[44]
*Meteorus pendulus* (Müller)	Hymenoptera	Braconidae	[44]
*Meteorus pulchricornis* (Wesmael)	Hymenoptera	Braconidae	[44]
*Meteorus versicolor* (Wesmael)	Hymenoptera	Braconidae	[44]
*Monodontomerus aereus Walker	Hymenoptera	Torymidae	[44]
*Ooencyrtus kuvanae* (Howard)	Hymenoptera	Encyrtidae	[44]
*Ooencyrtus masii* (Mercet)	Hymenoptera	Encyrtidae	[44]
*Parasarcophaga uliginosa* (Kramer)	Diptera	Sarcophagidae	[44]
*Parasetigana silvestris* (Robineau-Desvoidy)	Diptera	Tachinidae	[44]
*Pediobius cassidae* Erdös	Hymenoptera	Eulophidae	[44]
*Pediobius crassicornis* (Thomson)	Hymenoptera	Eulophidae	[44]
*Pediobius foliorum* (Geoffroy)	Hymenoptera	Eulophidae	[44]
*Pediobius pyrgo* (Walker)	Hymenoptera	Eulophidae	[44]
*Peribaea tibialis* (Robineau-Desvoidy)	Diptera	Tachinidae	[44]
*Perilampus neglectus* Bouček	Hymenoptera	Perilampidae	[44]
*Perilampus ruficornis* Fabricius	Hymenoptera	Perilampidae	[44]
*Phobocampe lymantriae* Gupta	Hymenoptera	Ichneumonidae	[44]
*Phobocampe unicincta* (Gravenhorst)	Hymenoptera	Ichneumonidae	[44]
*Pimpla disparis* Viereck	Hymenoptera	Ichneumonidae	[44]
*Pimpla rufipes* (Miller)	Hymenoptera	Ichneumonidae	[44]
*Pimpla turionellae* (L.)	Hymenoptera	Ichneumonidae	[44]
*Pronotalia carlinarum* (Szelényi and Erdös)	Hymenoptera	Eulophidae	[44]
*Protapanteles fulvipes* (Haliday)	Hymenoptera	Braconidae	[44]
*Protapanteles liparidis* (Bouché)	Hymenoptera	Braconidae	[44]
*Protapanteles nigerrimus* (Roman)	Hymenoptera	Braconidae	[44]
*Senometopia separata* (Rondani)	Diptera	Tachinidae	[44]
*Siphona boreata* Mesnil	Diptera	Tachinidae	[44]
*Tachina magnicornis* (Zetterstedt)	Diptera	Tachinidae	[44]
*Tachina praeceps* Meigen	Diptera	Tachinidae	[44]
*Telenomus embolicus* Kozlov	Hymenoptera	Scelionidae	[44]
*Telenomus laevisculus* (Ratzeburg)	Hymenoptera	Scelionidae	[44]
*Telenomus longistriatus* Kozlov	Hymenoptera	Scelionidae	[44]
*Telenomus lymantriae* Kozlov	Hymenoptera	Scelionidae	[44]
*Telenomus macroceps* Szabó	Hymenoptera	Scelionidae	[44]
*Telenomus phaIaenarum* Nees	Hymenoptera	Scelionidae	[44]
*Telenomus tetratomus* (Thomson)	Hymenoptera	Scelionidae	[44]
*Tetrastichomyia clisiocampae* Ashmead	Hymenoptera	Eulophidae	[44]
*Tetrastichus* sp.	Hymenoptera	Eulophidae	[44]
*Theronia atalantae* (Poda)	Hymenoptera	Ichneumonidae	[44]
*Torymus anastativorus* Fahringer	Hymenoptera	Torymidae	[44]
*Tyndarichus kuriri* Fahringer	Hymenoptera	Encyrtidae	[44]
*Tyndarichus navae* Howard	Hymenoptera	Encyrtidae	[44]
*Zenillia libatrix* (Panzer)	Diptera	Tachinidae	[44]

## Data Availability

Data are contained within the article.
